# Oxidative stress‐induced structural changes in the microtubule‐associated flavoenzyme Irc15p from Saccharomyces cerevisiae

**DOI:** 10.1002/pro.3517

**Published:** 2018-12-17

**Authors:** Karin Koch, Emilia Strandback, Shalinee Jha, Gesa Richter, Benjamin Bourgeois, Tobias Madl, Peter Macheroux

**Affiliations:** ^1^ Institute of Biochemistry Graz University of Technology Graz Austria; ^2^ Gottfried Schatz Research Center for Cell Signaling, Metabolism and Aging, Molecular Biology and Biochemistry Medical University of Graz Graz Austria; ^3^ BioTechMed‐Graz Graz Austria

**Keywords:** oxidative stress, thiol modification, flavin adenine dinucleotide, lipoamide dehydrogenase, microtubule‐binding protein

## Abstract

The genome of the yeast Saccharomyces cerevisiae encodes a canonical lipoamide dehydrogenase (Lpd1p) as part of the pyruvate dehydrogenase complex and a highly similar protein termed Irc15p (increased recombination centers 15). In contrast to Lpd1p, Irc15p lacks a pair of redox active cysteine residues required for the reduction of lipoamide and thus it is very unlikely that Irc15p performs a similar dithiol‐disulfide exchange reaction as reported for lipoamide dehydrogenases. We expressed *IRC15* in Escherichia coli and purified the produced protein to conduct a detailed biochemical characterization. Here, we show that Irc15p is a dimeric protein with one FAD per protomer. Photoreduction of the protein generates the fully reduced hydroquinone without the occurrence of a flavin semiquinone radical. Similarly, reduction with NADH or NADPH yields the flavin hydroquinone without the occurrence of intermediates as observed for lipoamide dehydrogenase. The redox potential of Irc15p was −313 ± 1 mV and is thus similar to lipoamide dehydrogenase. Reduced Irc15p is oxidized by several artificial electron acceptors such as potassium ferricyanide, 2,6‐dichlorophenol‐indophenol, 3‐(4,5‐dimethyl‐2‐thiazolyl)‐2,5‐diphenyl‐2*H*‐tetrazolium bromide, and menadione. However, disulfides such as cystine, glutathione, and lipoamide were unable to react with reduced Irc15p. Limited proteolysis and SAXS‐measurements revealed that the NADH‐dependent formation of hydrogen peroxide caused a substantial structural change in the dimeric protein. Therefore, we hypothesize that Irc15p undergoes a conformational change in the presence of elevated levels of hydrogen peroxide, which is a putative biomarker of oxidative stress. This conformational change may in turn modulate the interaction of Irc15p with other key players involved in regulating microtubule dynamics.

**Abbreviations**
*A. vinelandii*: *Azotobacter vinelandii*
DCPIP: 2,6‐dichlorophenol‐indophenol
DTT: dithiothreitol
*E. coli*: *Escherichia coli*
Irc15: increased recombination centers 15
LPD: lipoamide dehydrogenase
MQ: menadione
MTT: 3‐(4,5‐Dimethyl‐2‐thiazolyl)‐2,5‐diphenyl‐2*H*‐tetrazolium bromide
ROS: reactive oxygen species
*S. cerevisiae*: *Saccharomyces cerevisiae*
*S. seoulensis*: *Streptomyces seoulensis*
STH: soluble pyridine nucleotide transhydrogenases

## Introduction

Reactive oxygen species (ROS), such as hydrogen peroxide (H_2_O_2_), superoxide anions (·O_2_^−^), and hydroxyl radicals (·OH) are constantly generated during aerobic respiration. Organisms employ various strategies to preserve an intrinsic balance in the overall redox environment within the cell by simultaneously producing low levels of ROS essential for physiological signaling processes. An imbalance between the production of ROS and the antioxidant defenses to eliminate these toxic intermediates can lead to oxidative damage to DNA, lipids, and proteins, generating cellular stress.^1^ In the recent past, an increasing number of proteins have been identified that use reversible ROS‐mediated thiol modifications to regulate their function. Similar to other post‐translational modifications, oxidative thiol modifications are fully reversible, the extent of which depend on the reactivity and accessibility of cysteine thiols and the concentration of ROS present. Under oxidative stress conditions in the cell, these thiol modifications can become irreversible, leading to deleterious effects on protein structure and function.^2^, ^3^, ^4^

In this study, we report an uncharacterized flavoprotein from the yeast *Saccharomyces cerevisiae*, which was found to be structurally sensitive to oxidative damage. The genome of *S. cerevisiae* features 68 genes that were identified to encode a flavoprotein. Despite being a widely utilized model organism biochemical information on the flavoproteome is rather limited. For example, Irc15p (increased recombination centers 15) has a sequence similarity of 59% to the FAD‐containing yeast lipoamide dehydrogenase 1 (Lpd1p).^5^ Although it was demonstrated that Irc15p is associated with microtubules and regulates their dynamics,^6^ it is currently unclear whether the protein carries a flavin cofactor not to mention the potential properties and function of the putative enzymatic activity. This lack of information prompted us to recombinantly produce Irc15p and study its properties.

Lipoamide dehydrogenases (LPDs) orchestrate the reversible transfer of electrons between dihydrolipoamide to the enzyme‐bound FAD cofactor and NAD^+^. Generally, LPDs possess a second redox active group that is composed of two cysteine residues capable of forming an internal disulfide. This internal dithiol‐disulfide exchange communicates the electrons between the lipoamide and the FAD cofactor and is thus an essential asset of LPDs.^7^, ^8^ LPDs also constitute a component of oxoacid dehydrogenases that are large multienzyme complexes. In these complexes, LPDs reoxidize the covalently bound lipoamide cofactor of the transacylase component.^9^ Interestingly, Irc15p lacks the two essential cysteines required for the formation of a disulfide and, therefore, it is most unlikely that Irc15p is a redundant LPD or even exhibits similar enzymatic properties. Apparently, *IRC15* evolved after the whole genome duplication of *S. cerevisiae* and the duplicated *LPD1* sequence subsequently evolved to attain a new function (“neofunctionalization”).^10^, ^11^ This new function appears to be connected to the regulation of microtubule dynamics and chromosome segregation.^6^ However, it is not known what exactly this function is let alone whether this function is compatible with the properties of a putative LPD homolog.

LPDs are members of the family of flavoprotein disulfide reductases that catalyze the NAD(P)H‐dependent reduction of disulfide containing substrates. To perform this reaction the enzymes are equipped with a flavin cofactor and another non‐flavin redox center. Initially, only three members, namely LPD, glutathione reductase, and thioredoxin reductase composed the enzyme family, which has expanded significantly in recent years.^7^ In 2012, the family was classified according to the nature and position of the non‐flavin redox center into five sub groups.^8^ Group one comprises the flavoprotein disulfide reductases with the classical sequence motif CXXXXC, such as LPD. Members of group two are structurally related but contain a second cysteine based redox center. Enzymes from group three contain only one cysteine, which either forms a cysteine sulfenic acid or a cysteine‐coenzyme A mixed disulfide during the reaction. Members of group four contain the classical sequence motif but catalyze a non‐disulfide reductase reaction. Finally, members of group five feature two cysteines that are widely separated in the primary sequence.

In addition to these five sub‐groups, several proteins described in the literature exhibit high sequence similarity with flavoprotein disulfide reductases, but lack some significant features. For example, pyridine nucleotide transhydrogenases (STH) catalyzes the reversible transfer of electrons between NADH and NADP^+^ and lack at least one of the redox active cysteines and a histidine residue essential for catalytic activity.^7^, ^12^ These enzymes are also closely related to LPD, for example, STH from *Escherichia coli* exhibits 27% identity and 45% similar to several LPDs.^13^ However, also LPDs themselves are able to catalyze transhydrogenase reactions.^14^ Another example is LpdA from *Mycobacterium tuberculosis,* which lacks one cysteine as well as the catalytic histidine and glutamate. Like STH the protein is not able to catalyze the reduction of disulfides but instead features quinone reductase activity. The physiological relevance of this protein is unknown.^15^ In the present work, we recombinantly produced Irc15p in *E. coli*. The purified Irc15p shows the typical characteristics of a flavoenzyme. We have demonstrated that Irc15p is efficiently reduced by NADH but lacks disulfide reductase activity. However, reduced Irc15p reduces a range of artificial electron acceptors, such as potassium ferricyanide, 2,6‐dichlorophenol‐indophenol (DCPIP) and quinones. The potential role of Irc15p as a microtubule‐associated protein is discussed in light of our findings concerning the enzymatic properties and structural changes that occur upon exposure to hydrogen peroxide.

## Results

### Biochemical characterization of Irc15p

**Figure 1:**
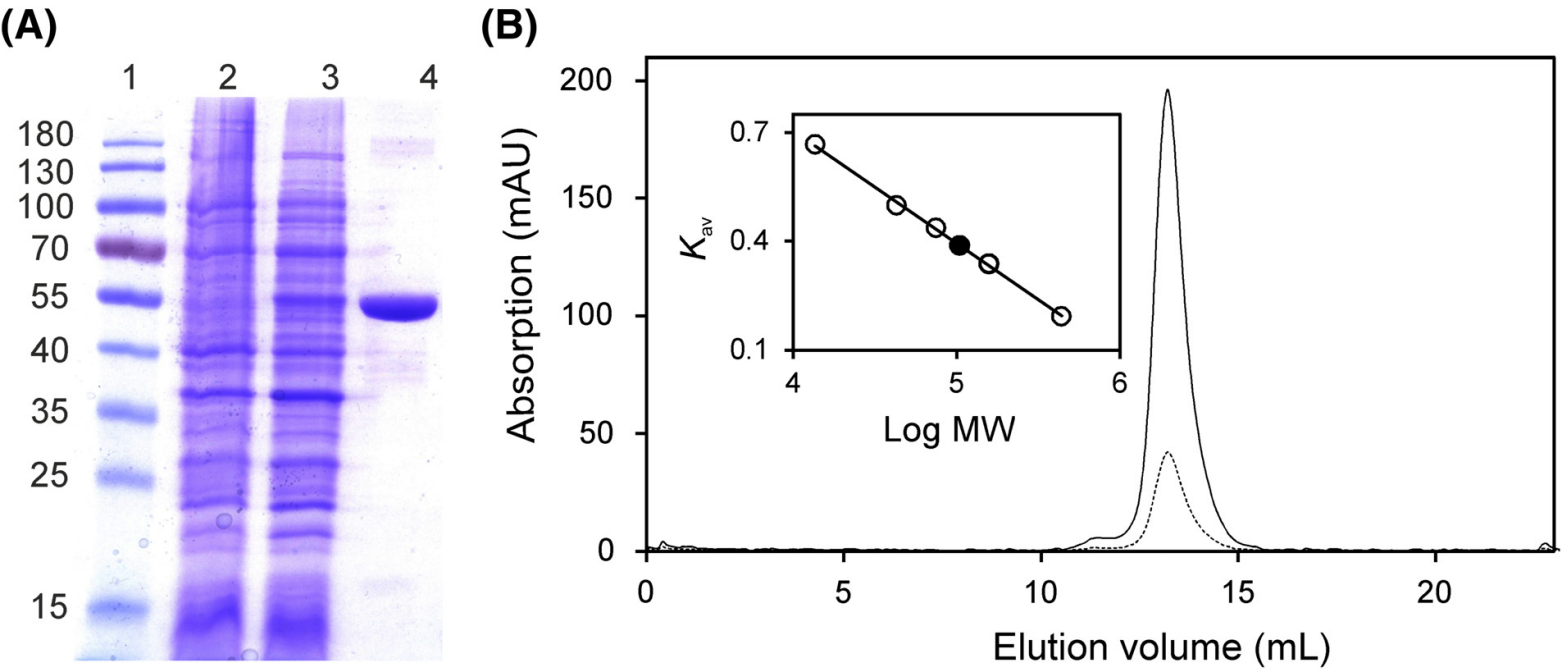
Determination of the purity and molecular mass of Irc15p using SDS‐PAGE and analytical size exclusion chromatography. (A) Determination of purity and subunit molecular mass of Irc15p by SDS‐PAGE after purification by affinity chromatography. Lane 1, PageRuler™ prestained protein ladder (10–180 kDa); Lane 2, protein extract before induction; Lane 3, protein extract after induction of *IRC15*; Lane 4, protein fraction after purification by Ni‐NTA‐sepharose. The subunit molecular mass of Irc15p was estimated to ~55 kDa. (B) Determination of native molecular mass of Irc15p (solid and dotted line display the absorption at 280 nm and 450 nm, respectively) using analytical size exclusion chromatography. The insert shows a plot of the partition coefficient (*K*_av_) against the logarithm of molecular mass of standard proteins (ferritin, 440 kDa; aldolase, 158 kDa; conalbumin, 75 kDa; ovalbumin, 43 kDa; ribonuclease A, 13.7 kDa). The calculated molecular mass of Irc15p (~ 113 kDa, black circle) indicates that Irc15p is present as a dimer.

Initially, Irc15p was produced with a C‐terminal hexa‐histidine tag as described by Keyes and Burke.^6^ However, the protein could not be purified successfully due to weak binding to the Ni‐NTA sepharose resin. Therefore, we employed a C‐terminal nona‐histidine tag enabling the successful purification of ~13 mg of protein from 1 L culture with a purity of >95% as judged by visual inspection of SDS‐PAGE and by using the program ImageJ (http://imagej.nih.gov/ij/)^16^ (Fig. 1, Panel A). The presence of DTT in the buffer was critical to prevent the precipitation of Irc15p.

Analytical size exclusion chromatography yielded a molecular mass of ~115 kDa confirming that Irc15p forms a homodimer as previously reported by Keyes and Burke.^6^ The protein peak was associated with a yellow color indicating the presence of a flavin cofactor in agreement with the high sequence similarity to the FAD‐dependent LPD (Fig. 1, Panel B).

**Figure 2:**
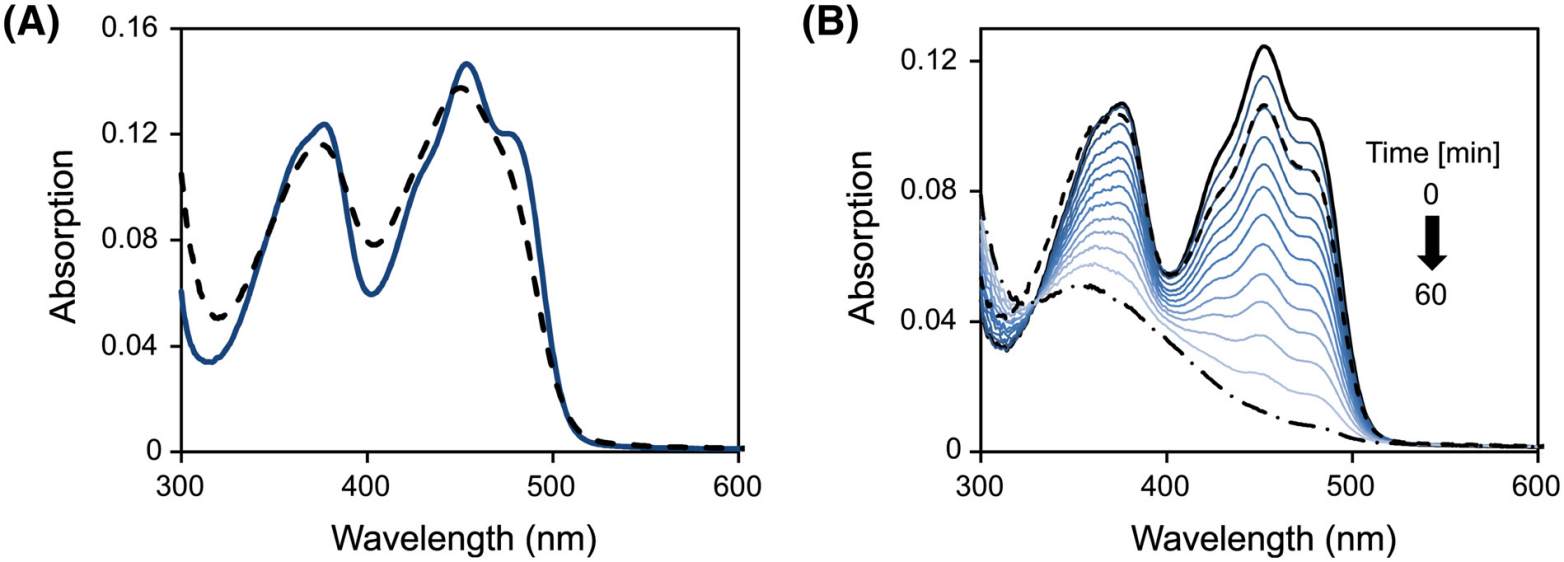
UV/Vis absorption spectroscopy. (A) UV–visible absorption spectrum of Irc15p before (solid line) and after denaturation (dashed line). Denaturation of purified Irc15p was carried out in Buffer B (50 mM HEPES, 50 mM NaCl, 1 mM DTT, pH 7.0) containing 0.2% SDS. (B) Absorption spectra observed during the anaerobic photoreduction of Irc15p in 50 mM HEPES, 50 mM NaCl, 1 mM DTT, 1 mM EDTA, pH 7.0. The solid black line represents the spectrum before irradiation. The reduction proceeds as indicated by the arrow with the dashed dotted line representing the final spectrum. After reoxidation by dioxygen the protein was partially denatured. The solution was cleared by centrifugation and the spectrum recorded (dashed line).

To assess the chemical identity of the flavin cofactor, Irc15p was denatured and the released flavin was analyzed by HPLC. A peak was obtained at a retention time of 9.1 min closely corresponding to the retention time of authentic FAD (9.05 min). Furthermore, the purified protein exhibited the absorption characteristics of a flavoprotein and also looks very similar to lipoamide dehydrogenases, with two distinct peaks at 377 and 453 nm with a shoulder at ~470 nm.^17^ Denaturation of the protein resulted in a slight bathochromic shift of the absorption maxima at 453 nm (Fig. 2, Panel A). Using an extinction coefficient of 11.300 M^−1^ cm^−1^ at 450 nm for free FAD^18^ an extinction coefficient of 11.900 M^−1^ cm^−1^ at 453 nm was calculated for Irc15p. This extinction coefficient was used to determine the concentration of Irc15p in further experiments. The *A*_280_/*A*_450_ ratios of purified Irc15p were usually between 4.3 and 4.5.

Photoreduction of Irc15p in the presence of EDTA led to the formation of the fully reduced flavin (hydroquinone) without the formation of a semiquinone radical (Fig. 2, Panel B). After reoxidation and removal of precipitated protein the obtained UV–vis absorption spectrum was similar to the initial spectrum indicating that reduction is fully reversible and does not give rise to chemical alterations of the flavin (Fig. 2, Panel B).

**Figure 3:**
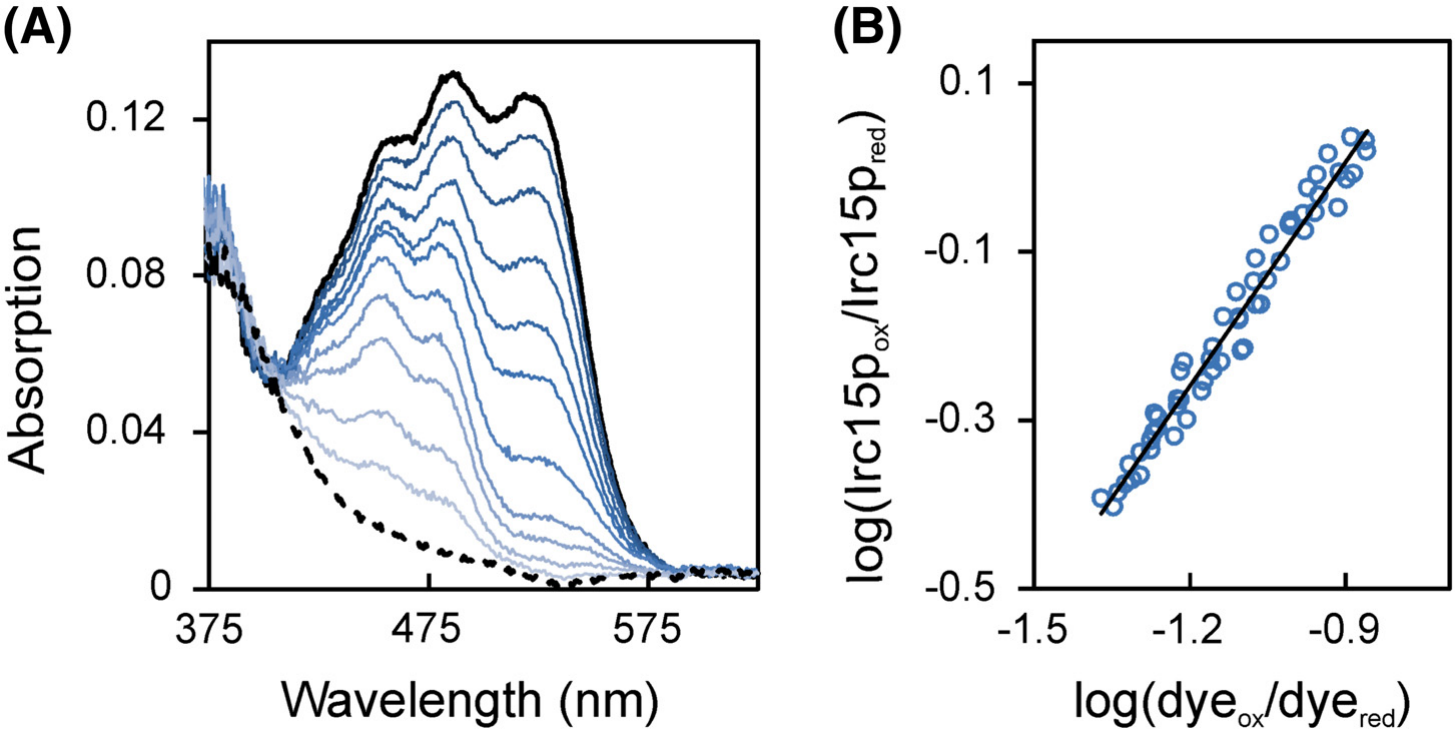
Redox potential determination of Irc15p in the presence of safranine T. (A) The absorption spectrum of the fully oxidized and fully reduced species are represented by a solid and dashed black line, respectively. Selected spectra of the course of reduction are represented in different shades of blue. 10 μM Irc15p was reduced by the xanthine/xanthine oxidase electron delivering system in the presence of safranine T over a time period of ~100 min. Data points for evaluation were extracted at 430 nm and 530 nm for Irc15p and for the dye safranine T, respectively. (B) Double logarithmic plot of the concentration of oxidized/reduced Irc15p vs. the concentration of oxidized/reduced safranine T (Nernst plot).

The redox potential of the FAD cofactor was determined with the xanthine/xanthine oxidase system in the presence of safranin T (*E*_M_ = −289 mV). According to the method of Minnaert^19^ a plot of log(Irc15p_ox_/Irc15p_red_) versus log(dye_ox_/dye_red_) was used to estimate the redox potential to −313 ± 1 mV (six independent measurements). In agreement with the photoreduction, reduction of Irc15p occurred without formation of a semiquinone and accordingly the slope of the logarithmic plot was close to unity indicating that the reference dye as well as the isoalloxazine moiety of the flavin took up two electrons (Fig. 3).

### Sequence alignment and homology modeling

**Figure 4:**
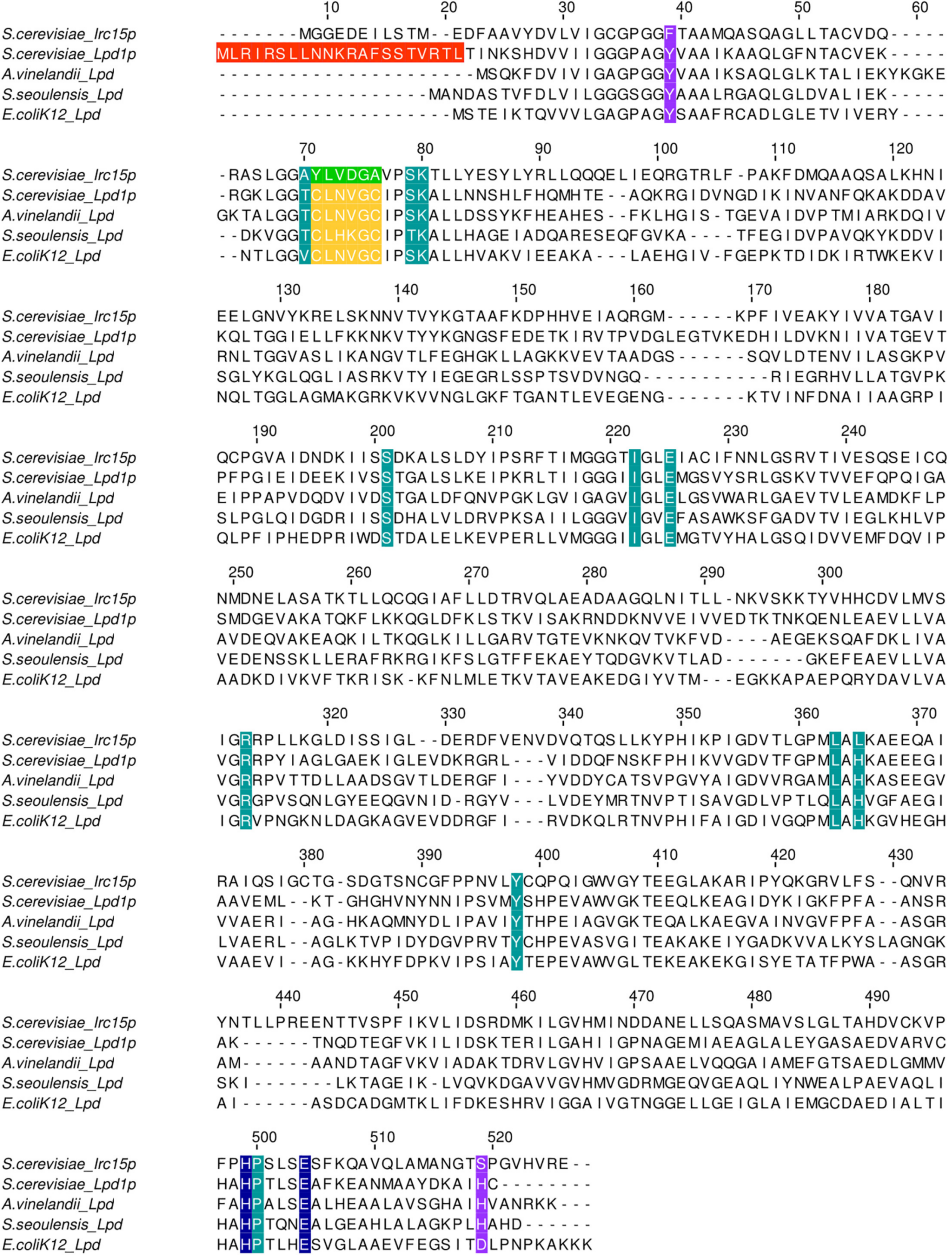
Alignment of the Irc15p protein sequence with sequences of LPD from *S. cerevisiae*, *E. coli*, *S. seoulensis and A. vinelandii*. The mitochondrial targeting sequence of Lpd1p is highlighted in red. The amino acid signature near the redox‐active disulfide is highlighted in yellow. The respective sequence in Irc15p is highlighted in green. The catalytic His‐Glu diad is highlighted in blue. Other residues in the active site are highlighted in petrol. Residues involved in structural stabilization are highlighted in purple.

A multiple sequence alignment using the amino acid sequence of Irc15p and the sequences of LPD from *S. cerevisiae*, *E. coli*, *Streptomyces seoulensis, and Azotobacter vinelandii* was generated (Fig. 4). The sequence identities of Irc15p and various LPDs are presented in Table 1.^20^, ^21^

**Table 1:** Sequence Identity of Irc15p in Comparison to Other LPDs from S. cerevisiae, E. coli, Streptomyces seoulensis and Azotobacter vinelandii

Percent identity to Irc15p	
*S. cerevisiae*_Lpd1p	40
*A. vinelandii*_Lpd	30
*S. seoulensis*_Lpd	28
*E. coli* K12_Lpd	27

**Figure 5:**
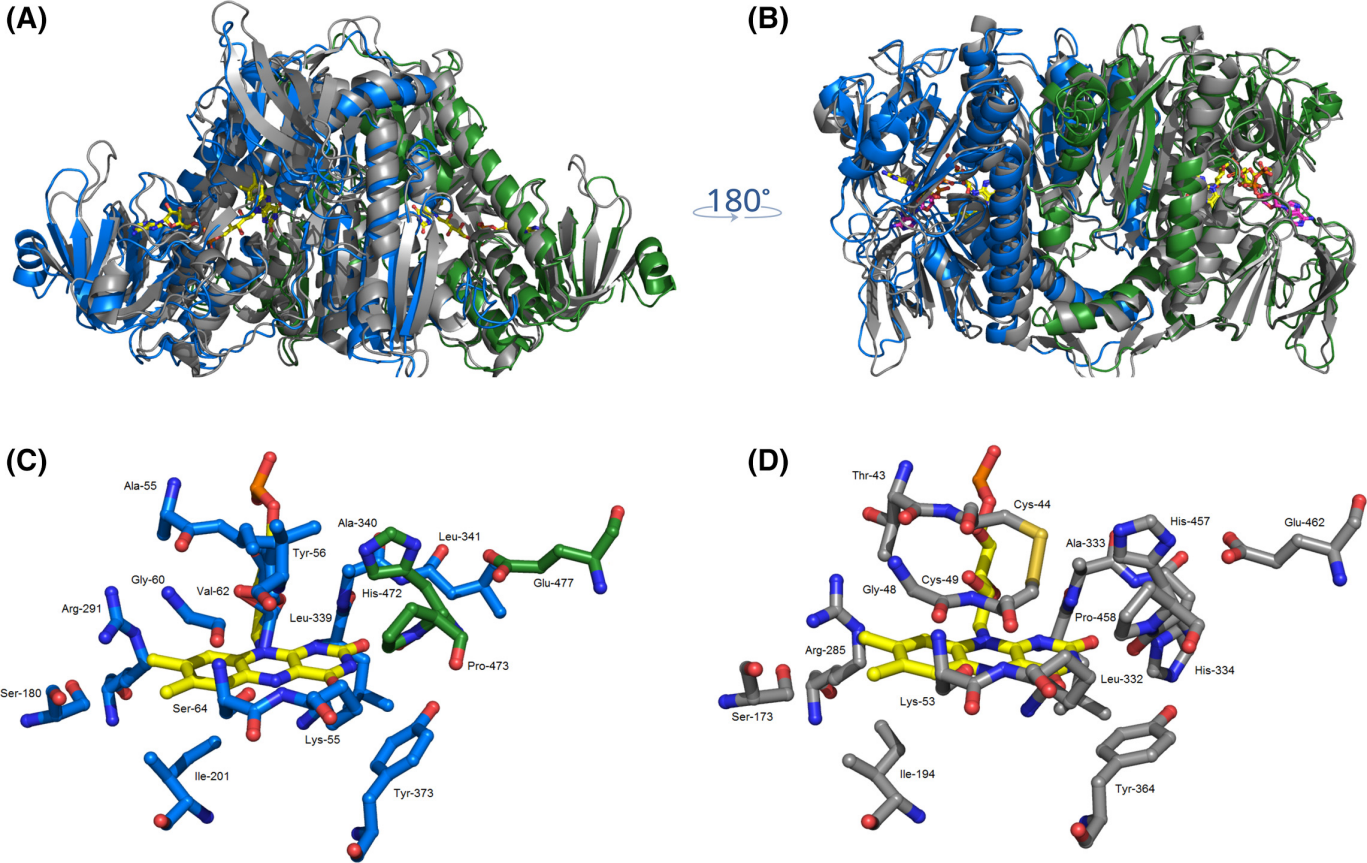
Overall structural similarity of Irc15p and LPD1p. (A) and (B) Structural superposition of LPD1p (grey, PDB code: 1V59) and Irc15p (blue/green). The FAD cofactor is displayed in yellow and NADH is shown in magenta. Close‐up view of the active sites of Irc15p (C) and LPD1p (D). Residues close to the FAD isoalloxazine ring are illustrated as grey sticks for both protomers (Lpd1p) or in colors corresponding to the respective protomer (Irc15p). Figures were prepared with the software PyMOL^25^.

A structural model of Irc15p was computationally generated using Lpd1p from *S. cerevisiae* (PDB entry: 1V59) as template (Fig. 5, Panels A and B).^22^, ^23^ A comparison of the close environment of the FAD cofactor from Irc15p (Fig. 5, Panel C) and Lpd1p (Fig. 5, Panel D) reflects a high sequence conservation: out of 16 residues that are within 4 Å of the flavin isoalloxazine ring only four are different. Notably, among these are the two cysteines, C44 and C49, which make up the dithiol/disulfide redox center of Lpd1p. These are replaced by tyrosine and alanine. The two amino acid residues that compose the catalytic diad, that is, H457 and E462 are conserved in both proteins.^17^

### Enzymatic properties and thermal stability of Irc15p

**Figure 6:**
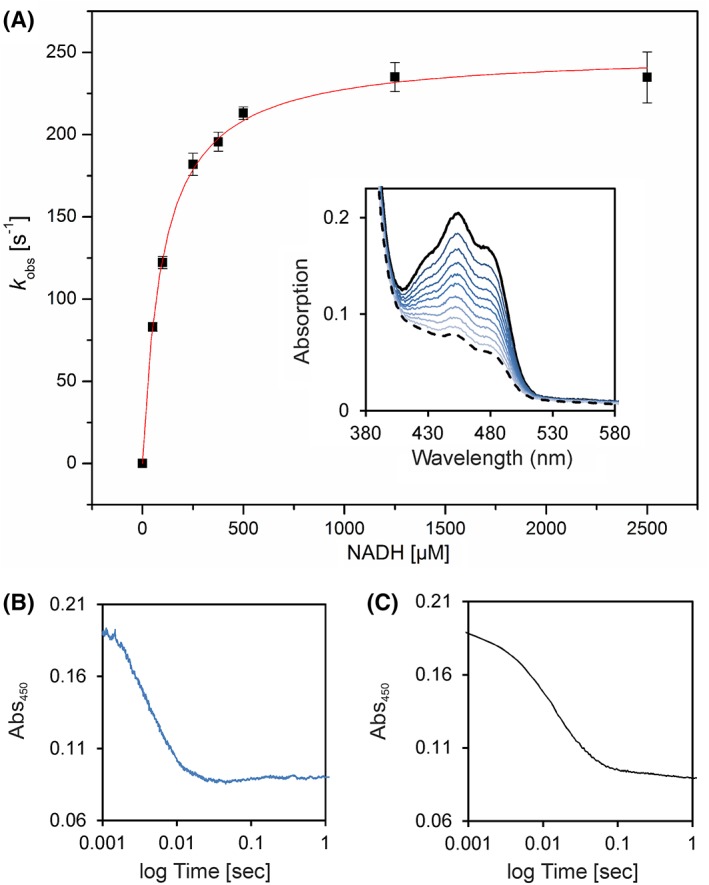
Pre‐steady‐state kinetics of Irc15p to determine reductive rates for NADH. (A) The rate of reduction was determined under anoxic conditions with the stopped flow device equipped with a diode array detector. At least three independent measurements were performed (error bars are shown as standard deviations). The inset displays selected absorption spectra of the reduction of ~20 μM Irc15p with 375 μM NADH. (B) Absorption change at 450 nm of the reduction of ~20 μM Irc15p with 1250 μM NADH. (C) Absorption change at 450 nm of the reduction of ~20 μM Irc15p with 1000 μM NADPH.

To gain information on the specificity of the electron donor, the reductive half‐reaction was studied using stopped‐flow spectrophotometry. Reduction of Irc15p with NADH was fast and monophasic. The rate of reduction was analyzed as a function of substrate concentration and fitted to a hyperbolic equation yielding a limiting reductive rate of 250 ± 3 s^−1^ and a dissociation constant of 100 ± 5 μM (Fig. 6, Panel A). As noted above, no semi‐quinone radical was observed (Fig. 6, Panel A, inset). In contrast to reduction by NADH, the reduction with NADPH exhibited two phases (Fig. 6, compare Panels B and C) and the bimolecular rate constant determined at 100 μM NAD(P)H is an order of magnitude lower (NADH = 1.2×10^6^ M^−1^·s^−1^, NADPH = 3.4×10^5^ M^−1^·s^−1^).

To evaluate the enzymatic activity of Irc15p, assays with NAD(P)H and several potential electron acceptors were performed (Table 2). No activity was observed with disulfides such as lipoic acid, glutathione or cystine. On the other hand, the enzyme showed diaphorase activity with the non‐specific electron acceptors potassium ferricyanide, 3‐(4,5‐dimethyl‐2‐thiazolyl)‐2,5‐diphenyl‐2*H*‐tetrazolium bromide (MTT), and 2,6‐dichlorophenol‐indophenol (DCPIP) and quinone reductase activity with menadione (MQ). Furthermore the reduced cofactor was reoxidized by molecular oxygen, however, at a comparatively sluggish rate. A clear preference for NADH as electron donor is only observed in steady‐state assays employing potassium ferricyanide as electron acceptor.

**Table 2:** Specific Activities with Standard Deviations of Irc15p with NAD(P)H [μmol/min^−1^/mg^−1^] as Electron Donor in Comparison with the Specific Activity of LPD from *S. seoulensis* 26 and LPD from *S. cerevisiae* (in brackets)^27^ with NADH. Reduction of Ferricyanide, DCPIP, and MTT was Determined at *420, 600*, and *578 nm*, Respectively. All Other Reactions were Monitored at *380 nm*

Substrate	Specific activity with NADH	Specific activity with NADPH	Specific activity of LPD with NADH
	[μmol/min^−1^/mg^−1^]^a^	[μmol/min^−1^/mg^−1^]^a^	[μmol/min^−1^/mg^−1^]^c^
Ferricyanide	179.5 ± 3.41	17.9 ± 1.14	7.87 (1670.0^d^)
DCPIP^b^	3.88 ± 0.14	4.72 ± 0.22	61.4 (2.0^d^)
MQ*^,^ b	19.7 ± 0.82	19.3 ± 2.12	7.18
MTT*	1.62 ± 0.08	1.28 ± 0.02	‐
Lipoic acid*	0	0	15.6
Cystine*	0	0	0.80
Glutathione*	0	0	0.18
Oxygen*	1.0 ± 0.02	1.0 ± 0.02	0

a The reaction mixture for the measurements of Irc15p contained 50 mM HEPES, pH 7.0, 50 mM NaCl, 10 nM DTT, 500 μM NAD(P)H, and 500 μM electron acceptors (except MQ and DCPIP).

b The concentration of DCPIP and MQ were 50 and 200 μM, respectively.

c The reaction mixture for the measurements of LPD from *S. seoulensis* contained 50 mM sodium phosphate buffer, pH 7.4, 0.3 mM substrates, and 0.2 mM NADH.

d The reaction mixture for the potassium ferricyanide assay of LPD1 from *S. cerevisiae* contained 165 mM sodium acetate, pH 4.8, 0.7 mg/mL bovine serum albumin, 1 mM EDTA, 600 μM NADH, 670 μM potassium ferricyanide. The DCPIP assay contained phosphate buffer, pH 7.2, 0.7 mg/mL bovine serum albumin, 1 mM EDTA; 600 μM NADH, and 40 μM DCPIP.

The potassium ferricyanide assay was further used to determine the influence of various pH values and salt concentrations on the enzymatic activity of Irc15p. The highest activity was observed at pH 7.0 without salt in the assay buffer. Below and above pH 7.0, the activity is reduced by about 14–49%, and the presence of 150 mM NaCl decreased the activity at pH 7.0 by 40%.

The thermal stability of Irc15p was monitored using a thermal shift assay, performed with the fluorescent dye SYPRO® Orange.^24^ Under optimal conditions, Irc15p displays a rather high thermal stability of about 70 °C (Table 3).

**Table 3:** Activity and Thermal Stability of Irc15p at Various pH and in the Absence and Presence of NaCl. Melting Points are Given as the Average of Two Independent Measurements

Buffer composition	Activity [%]	T_m_ [°C]
50 mM HEPES, pH 6.0	51	70
50 mM HEPES, pH 7.0	100	69
50 mM HEPES, pH 8.0	86	62
50 mM Tris/HCl, pH 9.0	46	56
50 mM HEPES, pH 7.0, 150 mM NaCl	61	69

The reaction mixture for the activity assay contained also 10 nM DTT, 500 μM NADH, and 500 μM ferricyanide.

Additionally, measurements were performed in the presence and absence of NADH, NAD^+^, NADPH and NADP^+^, as summarized in Table 4. Interestingly, a significant decrease in melting temperature could be observed after addition of an excess of NADH or NADPH.

**Table 4:** Thermal Stability of Irc15p in 50 mM HEPES, 50 mM NaCl, 1 mM DTT, pH 7.0 in the Presence and Absence of 50 mM NADH, 50 mM NAD^+^, 50 mM NADPH, 50 mM NADP^+^, and 50 mM Sodium Dithionite. Melting Points are Determined as the Average of Two Independent Measurements

Condition	*T*_m_ [°C]
Control	68
NADH	47
NAD^+^	64
NADPH	45
NADP^+^	68
Sodium dithionite	65

### Limited proteolysis

**Figure 7:**
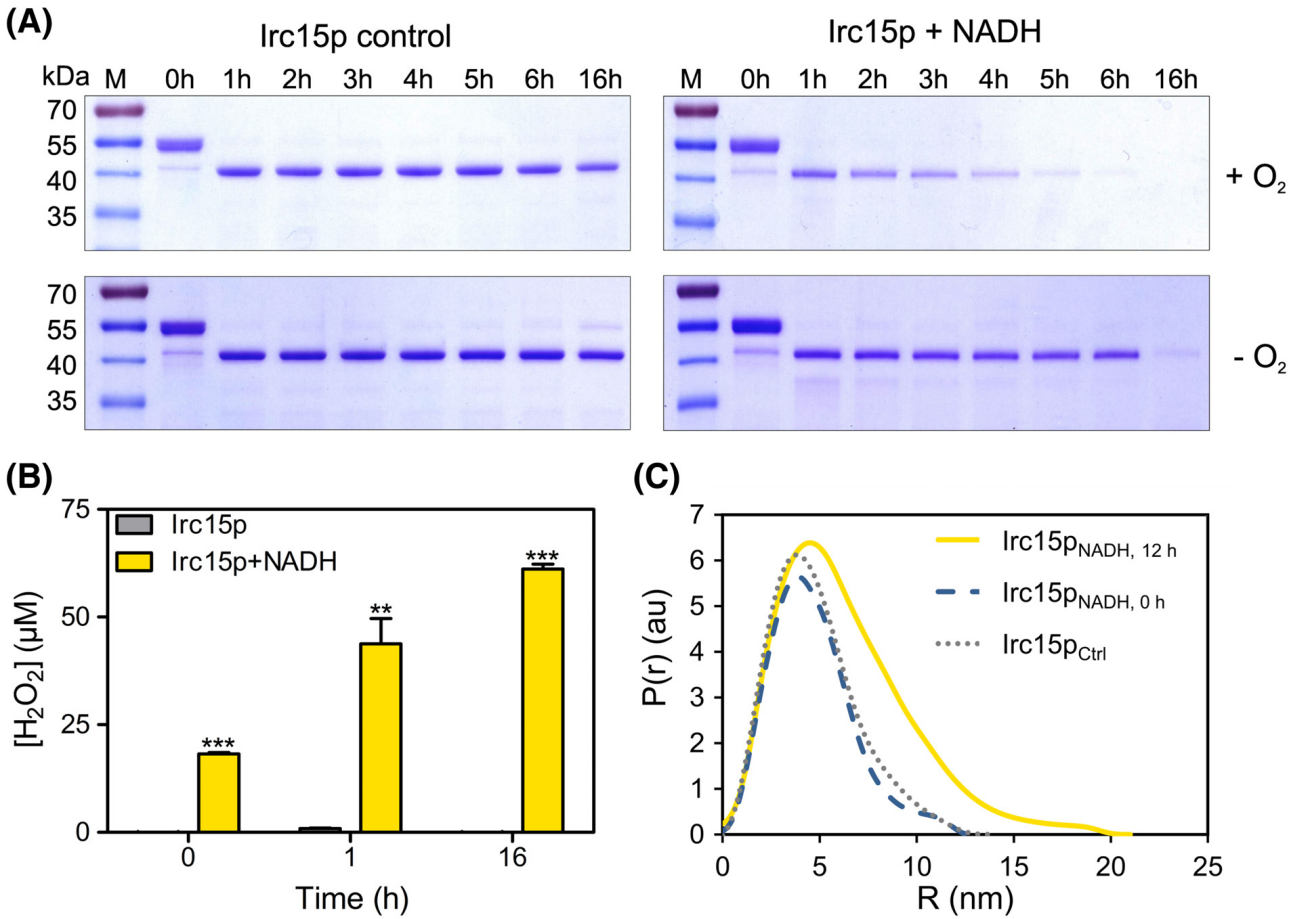
Limited proteolysis, hydrogen peroxide formation and SAXS data for Irc15p in the presence and absence of NADH. (A) SDS‐PAGE from the limited proteolysis experiment illustrating the effect of NADH and oxygen on the stability of the protein. Each gel has the marker PageRuler™ prestained protein ladder in Lane 1, the remaining lanes display the samples incubated for 0–16 h. (B) Hydrogen peroxide formation in Irc15p over time (0, 1, and 16 h) and in the presence and absence of NADH. (C) SAXS data comparing the experimental radial density distribution (P(r)) of Irc15p incubated with NADH measured after 0 and 12 h compared with a control sample without NADH.

To further study the effect of NAD(P)H on the protein stability, limited proteolysis using tryptic digestion was performed under oxic and anoxic conditions in the presence and absence of NADH. As displayed in Fig. 7A, Irc15p is more sensitive to proteolysis in the presence of NADH and molecular oxygen showing degradation already after 1 h whereas the control sample is stable overnight. Interestingly, when the same experiment was performed under anoxic conditions, no degradation was detectable after 6 h and became apparent only after 16 h.

### Hydrogen peroxide production

Since our limited proteolysis experiments demonstrated that NADH and molecular oxygen play a synergistic role, we hypothesized that the reduction of Irc15p by NADH and subsequent reoxidation by dioxygen led to the generation of hydrogen peroxide, which in turn may oxidize cysteine residues accessible on the surface of the protein. To assess this, we determined the production of hydrogen peroxide over a period of time by Irc15p in the presence of NADH under aerobic conditions. As expected, there was a marked increase in the level of hydrogen peroxide in the presence of NADH. In contrast, Irc15p without NADH showed no significant peroxide production (see Fig. 7B).

### SAXS measurements of Irc15p

In order to further investigate the potential impact of thiol oxidation on the overall structure of the protein, SAXS measurements were employed. Irc15p formed a dimer in solution with a radius of gyration (*R*_g_) of 3.64 ± 0.02 and with a maximum distance (*D*_max_) of 14 nm. However, incubation of Irc15p with NADH in the presence of molecular oxygen resulted in a substantial change of the radius of gyration (*R*_g_) and of the maximal diameter (*D*_max_) indicating that the dimer adopts a more extended conformation over a period of time (Irc15p with NADH measured directly: *R*_g_ = 3.56 ± 0.01, *D*_max_ = 13 nm, Irc15p with NADH measured after 12 h: *R_g_* = 5.44 ± 0.03, *D*_max_ = 21 nm, Fig. 7, Panel B). The SAXS data implicate that the presence of NADH is extending the conformation of Irc15p by 60%.

## Discussion

In this study, we have demonstrated that Irc15p is a flavoprotein with FAD as the cofactor. Recombinant Irc15p features characteristic spectral properties that are similar to those reported for LPDs (Fig. 2, Panel A). In contrast to LPDs, reduction of Irc15p does not give rise to the typical red charge transfer absorption at longer wavelength (~530 nm) owing to the lack of a pair of redox active cysteines near the FAD cofactor.^26^, ^28^ Instead, reduction by light as well as with NAD(P)H yielded the fully reduced FAD hydroquinone without the occurrence of a semiquinone radical (Fig. 2, Panel B and Fig. 6).

The redox potential determined for Irc15p is shifted by 93 mV to −313 ± 1 mV compared with free flavin (= −220 mV). The redox potentials determined for LPD from *E. coli* were *E*_ox_/EH_2_ = −264 mV and EH_2_/EH_4_ = −314 mV and were assigned to the redox potentials of the disulfide/dithiol and the FAD/FADH_2_ couple, respectively^28^. In order to confirm this assignment, Hopkins et al.^29^ created two variants lacking either one of the two participating thiol groups, that is, the variants C44S and C49S. The redox potentials of these variants were − 379 and − 345 mV, respectively, suggesting that the more negative redox potential determined for wild‐type LPD belongs to the FAD/FADH_2_ couple. Thus, the redox potential of the FAD/FADH_2_ couple of Irc15p is very similar to that of LPD suggesting that the environment of the FAD cofactors in these proteins is comparable.

Furthermore, we have demonstrated that the thermal stability of the protein is rather high (*T*_m_ = 70 °C). This is not unusual as the reported melting temperature for LPD from *A. vinelandii* is even higher (*T*_m_ = 80 °C).^30^ Interestingly, it was shown that an exchange of Y16 to phenylalanine leads to a decrease of the melting temperature to 72 °C, since Y16 stabilizes the interaction of the subunits via hydrogen bond formation to H470.^31^ In Irc15p phenylalanine is found in position 16 and H470 is replaced by serine. Therefore, the lower melting temperature of Irc15p may be accounted for, at least in part, by the amino acid changes in these positions.

To determine the substrate specificity of Irc15p the reductive half reaction was investigated using either NADH or NADPH. These measurements established a clear preference for NADH as electron donor proceeding with a limiting rate of *k*_red_ = 250 s^−1^ (Fig. 6). Thus, the limiting rate of Irc15p is an order of magnitude lower than that of lipoamide dehydrogenase (250 s^−1^ vs. >3000 s^−1^).^32^, ^33^ Since Irc15p is associated with microtubules and was shown to regulate their dynamics, the rapid reduction by NADH and the obvious conformational change occurring in the presence of NADH sheds new light on its potential role in processes such as mitosis. In recent years, several studies concluded that the NAD^+^/NADH ratio and the overall redox status are regulatory elements of the cell cycle and the dynamics of the cytoskeleton.^34^, ^35^, ^36^ It was shown that the NAD^+^/NADH ratio is high during the G0 phase, decreases during the S phase before it increases again in the G2 phase. However, no information is available of the NAD^+^/NADH ratio during mitosis.^35^ Furthermore, it has been demonstrated that NAD^+^ has an influence on the stability and curvature of microtubules. Since it is not interacting directly with the polymer it has been proposed, that NAD^+^ affects microtubule binding proteins on the plus‐end of the polymer.^36^ How exactly the redox state influences the cell cycle and the cytoskeletal dynamics is not known, but several proteins regulating the cell cycle as well as tubulin contain redox sensitive elements like cysteines or cofactors where modifications may occur.^37^, ^38^ Therefore, it is conceivable that reduced Irc15p interacts with these proteins and reduces oxidized groups, for example disulfides, to enable for example the polymerization of tubulins. This reactivity would clearly fit to the mode of action found in LPDs, where an internal disulfide in proximity to the isoalloxazine moiety of FAD is reduced to the dithiol via reduction of the flavin by NAD(P)H. In search for such an activity we tested a variety of disulfides, such as cystine, glutathione and lipoamide but we were unable to detect any reduction of these compounds (Table 2). However, we discovered that artificial electron acceptors such as potassium ferricyanide, DCPIP, MTT and MQ were good to excellent electron acceptors (Table 2).

A similar observation was reported for LpdA from *Mycobacterium tuberculosis*, which contains five homologs of flavoprotein disulfide reductases.^7^ Apparently, LpdA does not reduce disulfide containing compounds but similar to Irc15p reduces quinones.^7^ Interestingly, LpdA lacks one of the two cysteines near the FAD and the catalytic His‐Glu diad, in other words it shares the absence of the dithiol‐disulfide redox center with Irc15p but on the other hand also lacks the catalytic diad, which is present in Irc15p. Since the catalytic diad is important in the oxidative half reaction of disulfide reductases, that is, the formation of the internal disulfide by oxidation through the external disulfide (the “dithiol‐disulfide exchange reaction”), its presence in Irc15p suggests that it has retained the ability to catalyze a similar reaction.

Limited proteolysis experiments performed in the presence and absence of NADH under oxic and anoxic conditions showed that Irc15p became more susceptible to degradation in the presence of NADH and oxygen. Furthermore, we showed that the formation of hydrogen peroxide was responsible for the increased sensitivity toward tryptic digestion. In keeping with this, SAXS measurements for Irc15p indicated time‐dependent conformational change in Irc15p that resulted in a more extended and possibly more flexible structure. This structural change can be attributed to the presence of 11 cysteine residues per subunit of Irc15p, many of which are present on the surface. Cysteine is the most reactive and oxygen‐sensitive amino acid due to the presence of the side chain thiol group. ROS‐mediated oxidation of these thiols involves formation of sulfenic, sulfinic and sulfonic acids. While the sulfenic intermediates can be re‐converted to their reduced form, thereby modulating protein activity, the sulfinic and sulfonic acid states are irreversible in nature and can cause decreased protein stability. This phenomenon is called hyperoxidation and can be induced during oxidative stress.^39^ NADH‐mediated generation of H_2_O_2_ shown here mimics oxidative stress conditions in the yeast cell, where the excessive ROS accumulation may result from a plethora of sources such as electron leakage originating in the mitochondrial transport chain, hyperoxia, upregulation of certain enzymes such as D‐amino acid oxidases and peroxisomal acyl‐coenzyme A oxidases, xenobiotics and environmental factors such as heat stress.^1^ The study presented here showed that the yeast flavoprotein Irc15p is susceptible to redox‐regulated conformational change, which can potentially impair its interaction with tubulin leading to a negative regulation of the microtubule dynamics.^6^

## Materials and Methods

### Materials

All chemicals, reagents and enzymes were of highest quality and from Sigma‐Aldrich (St. Louis, USA), Roth (Karlsruhe, Germany) or Thermo Fisher Scientific (Waltham, USA), unless otherwise noted. Columns for affinity chromatography (Ni‐NTA‐sepharose), size exclusion chromatography (Superdex 200 10/300 GL) and buffer exchange (PD‐10 desalting column) were from GE Healthcare (Little Chalfont, UK). The *E. coli* strains Top10 and Rosetta™ (DE3) were from Invitrogen (Carlsbad, USA) and Merck (Darmstadt, Germany), respectively. The plasmid pET21d was from Merck (Darmstadt, Germany).

### Cloning of IRC15 for large scale expression in E. coli

All strains were generated using standard genetic techniques^40^, ^41^. Briefly, genomic DNA from *S. cerevisiae* was extracted with the yeast DNA extraction kit from VWR (Radnor, USA). According to the sequence for *IRC15* from the *Saccharomyces* genome database^41^ the following primers were designed and synthesized from VBC (Vienna, Austria): fw_5′‐GAACCATGGCAATGGGAGGTGAAGACGAAATATTAAGCAC‐3′; rev_5′‐GAGCCTCGAGTTAATGGTGATGATGGTGATGATGATGATGTTCCCGGACATGTACGCCAG ‐3′. To construct the heterologous expression vector pET21d(+)*IRC15* introducing an additional C‐terminal 9x‐histidine tag the restriction enzymes NcoI/XhoI were used. Individual clones were sequenced before transforming the plasmid into *E. coli* Rosetta™ (DE3) cells.

### Heterologous production and purification of Irc15p

A single colony of *E. coli* Rosetta (DE3) comprising pET21d(+)*IRC15* was used to inoculate a pre‐culture that was aerobically incubated (37 °C, 16 h, 150 rpm) in terrific broth media (bacto‐tryptone 12 g/L, bacto‐yeast extract 24 g/L, glycerol 4 g/L, KH_2_PO4 2.31 g/L, and K_2_HPO_4_ 12.54 g/L) supplemented with 100 μg·mL^−1^ ampicillin and 20 μg·mL^−1^ chloramphenicol. 1% pre‐culture was used to inoculate the main‐culture supplemented with 100 μg·mL^−1^ ampicillin and 10 μg·mL^−1^ chloramphenicol, which was incubated aerobically at 37 °C with agitation at 150 rpm until an *OD*_600_ of ~0.6 was reached. Production of the recombinant protein was induced by addition of 0.5 mM isopropyl‐β‐D‐thiogalactoside and the culture was further incubated for 16 h at 20 °C. Cells were harvested by centrifugation at 4.500*g* at 4 °C and washed once with 1% saline solution. Cell pellets were resuspended in 4 mL/g pellet buffer A (50 mM HEPES, 150 mM NaCl, 1 mM dithiothreitol, pH 7.0) supplemented with 30 mM imidazole, 1 mM phenylmethylsulfonyl fluoride dissolved in dimethylsulfoxide, 10 μM flavin adenine dinucleotide disodium salt hydrate. Furthermore, 1 μL of protease inhibitor cocktail for the purification of histidine‐tagged proteins from Sigma‐Aldrich (St. Louis, USA) was added per 1 g of cell pellet. Cell disruption was achieved by sonication with a Labsonic L instrument from Braun Biotech International (Berlin, Germany) with 120 Watt for 3 × 3 min in an ice‐water bath with 3 min pauses between each cycle. The cell lysate was centrifuged at 38.850 *g* for 45 min at 4 °C, and the supernatant was loaded onto a 5‐mL HisTrap HP column previously equilibrated with buffer A supplemented with 30 mM imidazole. The column was washed with five column volumes with buffer containing 50 mM HEPES, pH 7.0, 150 mM NaCl, 1 mM DTT and 100 mM imidazole. Then the column was washed with buffer A supplemented with 100 mM imidazole and subsequently proteins were eluted with buffer A supplemented with 350 mM imidazole. Fractions containing target protein were pooled and concentrated with centrifugal filter units (Amicon Ultra‐15, 50 kDa; Millipore, Massachusetts, USA). Concentrated protein was re‐buffered to buffer B (50 mM HEPES, 50 mM NaCl, 1 mM DTT, pH 7.0) with a PD‐10 desalting column. The protein solutions were shock frozen and stored at −80 °C if not used immediately.

### Determination of molecular mass of Irc15p

The subunit molecular mass of purified Irc15p was determined by SDS‐PAGE with a 12.5% separating gel and 5% stacking gel under reducing conditions described by Laemmli.^43^ The molecular mass marker PageRuler™ (prestained protein ladder 10–180 kDa) from Thermo Fisher Scientific (Waltham, USA) was used.

To determine the native molecular mass of Irc15p size exclusion chromatography with Buffer A using a Superdex 200 10/300 GL column attached to an Äktapurifier™ system from GE Healthcare (Little Chalfont, UK) was performed. Protein elution was monitored at 280 nm and 450 nm. The column was calibrated with molecular mass standards according to the instructions from GE healthcare.

### Determination of the flavin cofactor bound to Irc15p

To determine the nature of the flavin cofactor concentrated protein samples were treated with 8 M guanidine hydrochloride (pH 2 adjusted with concentrated HCl). Denatured protein was removed by centrifugation (13,000 *g*, 5 min) and the solution was neutralized with concentrated NaOH. To remove residual protein centrifugal filter units (Amicon Ultra‐0.5 mL 10 kDa; Millipore, Massachusetts, USA) were used. The flow‐through was concentrated at 50 °C under reduced pressure and subsequently analyzed by HPLC (UltiMate® 3000 HPLC system from Dionex, California, USA) using an Atlantis® dC18 5 μM (4.6 × 250 mm) column. As liquid phase a 0.1% TFA solution and acetonitrile containing 0.1% TFA were used. The concentration of the organic solvent was increased within 20 min from 0% to 95% in a linear gradient (*T* = 25 °C; flow rate = 1 mL/min). The samples were analyzed using a diode array detector at 280, 370, and 450 nm. The retention times of authentic FAD, FMN and riboflavin were 9.05, 9.75, and 10.4 min, respectively.

### Determination of the redox potential

The redox potential was determined by the dye‐equilibration method using the xanthine/xanthine oxidase electron delivering system as described by Massey.^44^ Reactions were carried out in buffer C (50 mM HEPES, 50 mM NaCl, pH 7) supplemented with methyl viologen (2.5 μM) as mediator, 500 μM xanthine, and xanthine oxidase in catalytic amounts (~40 nM) and lasted 0.5–2 h at 25 °C. The protein concentration for a typical experiment was ∼10 μM. The concentrations given are final values after mixing in the flow cell. Experiments were performed with a SF‐61SX2 stopped flow device from TgK Scientific Limited (Bradford‐on‐Avon, UK) equipped with an auto‐shutter to reduce photochemical effects during the experiment. To maintain anoxic conditions the device was positioned in a glove box from Belle Technology (Weymouth, UK). Absorption spectra during the course of reduction were recorded with a KinetaScanT diode array detector from TgK Scientific Limited (Bradford‐on‐Avon, UK). Safranin T was used as a reference dye for the analysis (−289 mV). The amounts of oxidized and reduced Irc15p and safranin T were quantitated at 430 nm and 530 nm, respectively. The reduction–oxidation potentials were calculated from plots of log(Irc15p_ox_/Irc15p_red_) versus log(dye_ox_/dye_red_) according to Minnaert^19^ using Excel 2010 (Microsoft, Redmond, WA, USA).

### Sequence alignment and homology modeling

A multiple sequence alignment was generated with the program Clustal Omega^21^ with sequences taken from the *Saccharomyces* genome database^41^ and from the UniProt database.^20^ A computational prediction approach was employed to construct the model structure of Irc15p. Tertiary structure of the protein was generated using protein homology‐based molecular modeling software Swiss‐Model^22^ and *ab initio* threading based software I‐TASSER^23^. Both the programs used Lpd1p from *S. cerevisiae* (PDB entry: 1V59) as the top threading template for automated model building.

### Methods using UV–visible absorption spectroscopy

Absorption spectra were recorded with a Specord 200 plus spectrophotometer from Analytik Jena (Jena, Germany) at 25 °C using 1‐cm quartz cuvettes.

*Extinction coefficient*: The extinction coefficient of Irc15p was determined according to Macheroux.^18^ Briefly, Irc15p bound FAD was released by addition of 0.2% SDS. Absorption spectra were recorded before and after denaturation of the enzyme. The calculation yielded an extinction coefficient of 11.900 M^−1^ cm^−1^ at 453 nm for Irc15p.

*Anoxic photoreduction:* Photoreduction was carried out as described by Macheroux.^18^ Briefly, ~10 μM Irc15p in 1 mL buffer B (50 mM HEPES, 50 mM NaCl, 1 mM DTT, pH 7.0) supplemented with 1 mM EDTA was deoxygenated by incubation for 2 h in a glove box from Belle Technology (Weymouth, UK). A 10 W LED floodlight (Luminea) was used to reduce the cofactor by light irradiation. Absorption spectra were recorded after each reduction step until no further spectral changes were observed. Thereafter the sample was exposed to air and a spectrum was recorded after complete reoxidation.

*Steady state kinetics:* Initial‐velocity kinetic measurements were performed in triplicates with NAD(P)H as an electron donor and the disulfide containing electron acceptors lipoic acid, glutathione and cystine and the artificial electron acceptors potassium ferricyanide, MQ, MTT and DCPIP. Reaction mixtures were setup in buffer C (50 mM HEPES, 50 mM NaCl, pH 7). All reactions were initiated by addition of 5 μL enzyme stock solution supplemented with 200 nM DTT to the reaction mixture – final enzyme concentrations were 10 nM. Controls were performed in the absence of enzyme. Rates of reduction with MQ, oxygen and disulfide containing substrates were determined by fitting the observed absorption change at 380 nm in the first minute using adapted extinction coefficients (NADH ε_380 nm_ = 1.210 M^−1^·cm^−1^ or NADPH ε_380 nm_ = 1.280 M^−1^·cm^−1^). For the other electron acceptors, pertinent wavelengths and extinction coefficients were used (ferricyanide ε_420 nm_ = 1.040 M^−1^·cm^−1^; MTT ε_578 nm_ = 13.000 M^−1^·cm^−1^; DCPIP ε_600 nm_ = 21.000 M^−1^·cm^−1^).^39^

### Thermal shift assay

Thermal shift assays were performed as described by Ericsson *et al*.^24^ 20 μL of ~13 μM Irc15p protein solution was pipetted into a white 96‐well RT‐PCR plate from Bio‐Rad (California, USA) both at different pH, in the absence and presence of 150 mM NaCl and in buffer B in the presence and absence of a final concentration of 50 mM NADH, 50 mM NAD^+^, 50 mM NADPH, 50 mM NADP^+^, or 50 mM sodium dithionite. Two μL of a 1:500 dilution of SYPRO® orange from Molecular Probes (Oregon, USA) was added. The plates were sealed with an Optical‐Quality Sealing Tape from Bio‐Rad (California, USA) and heated in a CFX Connect™ Real‐Time PCR detection system from Bio‐Rad (California, USA) from 20 to 95 °C in increments of 0.5 °C/5 s. Fluorescence changes of the dye were detected at a wavelength between 470 and 500 nm. Melting temperatures (*T*_m_) were determined using CFX Manager 3.0 software from Bio‐Rad (California, USA).

### Determination of kinetic rates

The protein was deoxygenated by incubation for 2 h in a glove box from Belle Technology (Weymouth, UK) kept in nitrogen atmosphere. The reductive half‐reaction was investigated by mixing protein (~20 μM) in buffer B with 25–2.500 μM NADH or 25–1.000 μM NAD(P)H. The concentrations given are final values after mixing in the flow cell. Experiments were performed with a SF‐61SX2 stopped flow device from TgK Scientific Limited (Bradford‐on‐Avon, UK) positioned in an anoxic glove box from Belle Technology (Weymouth, UK) at 4 °C. Changes in flavin absorption were followed with a PM‐61s photomultiplier from TgK Scientific Limited (Bradford‐on‐Avon, UK) at 453 nm.

### Limited proteolysis

12 μM Irc15p in buffer D (50 mM HEPES, 50 mM NaCl, 5 mM EDTA, and 1 mM DTT, pH 7.0) in the presence and absence of 50 mM NADH and under oxic or anoxic conditions was digested using 5 μg/mL trypsin from Promega (Madison, WI, USA). The reactions were also supplemented with 8 mM DTT. The reactions were performed at 37 °C. Reactions in the absence of dioxygen were conducted in a glove box from Belle Technology (Weymouth, UK) filled with nitrogen gas. After preincubation of trypsin at 37 °C for 15 min, the digestion was started and samples were taken out after different time points (0, 1, 2, 3, 4, 5, 6, and 16 h). The reactions were stopped by adding SDS sample buffer and the samples were boiled at 95 °C for 10 min. The samples were then analyzed by SDS‐PAGE with a 12.5% separating and 5% stacking gel.^45^, ^46^

### Hydrogen peroxide assay

A time‐dependent generation of H_2_O_2_ by Irc15p in the presence and absence of NADH was monitored using the Pierce™ Quantitative Peroxide Assay Kit (ThermoFischer Scientific). For the assay, 20 μM Irc15p in buffer C (50 mM HEPES, 50 mM NaCl, pH 7.0) was incubated with 50 mM NADH at room temperature. A sample without NADH, also incubated at room temperature, was used as a control. The reactions were terminated at 0, 1, and 16 h by addition of 10% TCA solution. Samples of this reaction mixture (10 μL) were added to 100 μL of the working reagent in a 96‐well plate and incubated for 20 min at room temperature. Working reagent was prepared according to the protocol specified in the kit (1 vol of reagent A in 100 vol of reagent B). Absorbance was recorded at 595 nm on a plate reader (FLUOStar Omega plate reader, BMG Labtech). The values were normalized to account for the intrinsic absorption of the working reagent. A standard curve containing 0–100 μM of H_2_O_2_ was prepared to determine the amount of H_2_O_2_ present in each sample.

### Small‐angle X‐ray scattering

For successful SAXS measurements, an additional purification step of Irc15p was needed. Therefore, Irc15p in buffer D (50 mM HEPES, 50 mM NaCl, 5 mM EDTA, and 1 mM DTT, pH 7.0) was purified by size exclusion chromatography on a Superdex 200 Increase 10/300 GL column from GE Healthcare (Little Chalfont, UK) connected to an ÄKTApurifier™ system (GE Healthcare, Little Chalfont, UK). The protein containing fractions were then collected, centrifuged and used for further sample preparation.

For the SAXS measurements, three separate reaction mixtures were prepared, including one control with a concentration of 119 μM Irc15p and two samples with a concentration of 61 μM Irc15p, measured after 0 and 12 h incubation with 50 mM NADH at 4 °C. Buffers for background corrections were also prepared from buffer D with either 119 μM or 61 μM FAD in the absence or presence of 50 mM NADH. All samples contained 8.3 mM DTT to prevent precipitation of Irc15p.

SAXS data for Irc15p were recorded with an in‐house SAXS instrument (SAXSspace, Anton Paar, Graz, Austria) equipped with a Kratky camera, a sealed X‐ray tube source and a Mythen2 R 1 K Detector (Dectris). Thereby Irc15p and the buffers for background subtraction where loaded via an ASX autosampler and measured in a flow cell. The scattering patterns were measured with a 180‐min exposure time (180 frames, each 1 min). Radiation damage was excluded on the basis of a comparison of individual frames of the 180‐min exposures, wherein no changes were detected. A range of momentum transfer of 0.010 < *s* < 0.63 Å^−1^ was covered (*s* = 4π sin(*θ*)/*λ*, where 2*θ* is the scattering angle, and *λ* is the X‐ray wavelength, in this case 1.5 Å.

Obtained SAXS data were processed using the SAXSanalysis package (Anton Paar, version 3.0) and analyzed using the ATSAS package (version 2.8.2, Hamburg, Germany). The data were desmeared using GIFT (PCG‐Software). The forward scattering (I(0)), the radius of gyration (*R*_g_), the maximum dimension (*D*_max_), and the interatomic distance distribution function (P(r)) were computed with GNOM.^47^ The masses of the solutes were evaluated based on their Porod volume.

## Author contributions

P.M. initiated the project; K.K., E.S., G.R., S.J., B.B., T.M., and P.M. designed the experiments and interpreted the data. K.K. and E.S. produced and purified Irc15p. K.K., E.S., and S.J. performed analytical, biochemical and enzymatic experiments. G.R. and B.B. performed SAXS measurements. K.K., E.S., S.J. G.R., B.B., and P.M. wrote the manuscript.
